# Protection reveals density-dependent dynamics in fish populations: A case study in the central Mediterranean

**DOI:** 10.1371/journal.pone.0228604

**Published:** 2020-02-03

**Authors:** Paco Melià, Renato Casagrandi, Antonio Di Franco, Paolo Guidetti, Marino Gatto

**Affiliations:** 1 Dipartimento di Elettronica, Informazione e Bioingegneria, Politecnico di Milano, Milano, Italy; 2 Dipartimento Ecologia Marina Integrata, Sede Interdipartimentale della Sicilia, Stazione Zoologica Anton Dohrn, Lungomare Cristoforo Colombo (complesso Roosevelt), Palermo, Italy; 3 Université Côte d’Azur, CNRS, UMR 7035 ECOSEAS, Nice, France; 4 CoNISMa, Consorzio Nazionale Interuniversitario per le Scienze del Mare, Roma, Italy; Aristotle University of Thessaloniki, GREECE

## Abstract

Casting light on how the interaction between protection and density dependence affects fish population dynamics is critical for understanding the effectiveness of marine protected areas (MPAs). We developed a framework based on nonparametric statistics, model selection and multi-model inference to contrast alternative hypotheses about the effect of density dependence on demographic dynamics under protected and unprotected conditions. We trialed it using a 12-year long time series of white seabream (*Diplodus sargus sargus*) population density within the no-take zone of Torre Guaceto MPA (Italy) and at two nearby unprotected locations. Then, we showed how the demographic models obtained can be used to assess the consequences of protection on population viability. Population dynamics were significantly influenced by fish density within the MPA and at one of the unprotected locations, where demography is possibly driven by directional recruitment subsidy from the MPA. The comparison of population growth rates within and outside the MPA suggests that in unprotected conditions the fishery may remove a fraction between 40 and 70% of the population each year. The population viability analysis pointed out that, while the probability that the population becomes depleted (i.e. undergoes a local, temporary quasi-extinction) is high in unprotected locations, it is negligible within the no-take zone of the MPA.

## Introduction

Density dependence has important consequences on how fish populations respond to the establishment of marine protected areas (MPAs). In particular, density-dependent mortality determined by predation and competition is a key compensatory mechanism damping down inter-annual variability of recruitment [1]. While population density in exploited conditions may be too low to provide evidence for resource limitation, competition for food or space may become important in protected populations, affecting life history traits such as life span, body growth, mortality and reproductive patterns, as well as inducing density-dependent emigration [2–4]. Failing to take into proper account the effects of density dependence may lead to unreliable assessments of the actual benefits of protection.

Density dependence is traditionally considered to affect mainly early life history stages [5]; however, it can affect mortality and body growth of adult fishes as well [4,6,7]. Species with low mobility and weakly density-dependent biological parameters are expected to derive the largest population increase within MPA borders [8,9], while species with higher mobility and stronger density dependence can also replenish nearby unprotected areas through adult spillover [10–12]. Casting light on how the interaction between protection and density dependence affects fish population dynamics is hence critical to understanding the role of MPAs [13] and is recognized as a major research gap in MPA science [14]. Experimental studies conducted so far to compare protected and exploited fish populations were not always able to provide unequivocal evidence of the direction in which life history traits respond to population density [14]. For instance, juvenile mortality in three seabream species (genus *Diplodus*) within and outside MPAs along the Mediterranean coasts of Spain, France and Italy increased significantly with fish density, but mortality and body growth rates did not differ significantly inside and outside protected zones [15,16].

As the causal chain linking population density, life history traits and the long-term behavior of population dynamics is not always easy to disentangle, time series analysis is often used as a means to estimate density dependence in population growth rate [17]. However, the adoption of this approach for marine fishes in MPAs can be hampered by the scarcity of sufficiently long time series (see [18] for a valuable exception). In cases where they are available, effectively detecting density dependence remains difficult because of the biasing effect of census error. Traditional tests relying on the detection of a relationship between population density and the logarithm of population growth rate are, in fact, prone to spuriously detecting density dependence, because the measurement error in density for a given year appears in the corresponding change in population density with equal magnitude but opposite sign [19]. Different approaches have been proposed to take the effect of census error explicitly into account and bypass the limitations of conventional methods [20], but most of them rely on rather stringent assumptions regarding the particular functional form of density dependence (such as the Gompertz-logistic function; see e.g. [21,22]). While a reliable identification of the most likely form of density dependence may not be particularly relevant when the main purpose of the analysis is simply to test for the presence or absence of a generic density-dependent mechanism, it becomes crucial when one wants to build a model that can be used as a predictive tool to assess the response of a population to different conservation or management strategies [23].

For these reasons, here we develop a framework aimed at investigating whether and how population density regulates demographic dynamics under protected and unprotected conditions. We use an empirical approach to time series analysis, based on model selection to contrast alternative (density-dependent and density-independent) models rather than relying on strong hypotheses about the functional form of the model. The proposed approach allows the explicit integration of available information on sampling uncertainty. We trial the framework using a 12-year long time series of abundance data of the sparid fish *Diplodus sargus sargus*, collected between 2004–2015 within and around an effectively enforced MPA (Torre Guaceto, south-western Adriatic Sea, Mediterranean). We then exemplify how the demographic models derived from the application of the framework can be used to evaluate the long-term viability of a population under protected and unprotected conditions.

## Materials and methods

### Study species

Seabreams belonging to the genus *Diplodus* are a valuable resource for small scale and recreational fisheries along Mediterranean coasts [24], and are therefore subjected to intense fishing pressure. However, they also play a key role in rocky shore ecosystems, where their protection is fundamental not only for the conservation of the species themselves, but also to preserve (or restore) community structure and ecosystem functioning [14,25,26]. The white seabream *Diplodus sargus sargus*, which inhabits infra-littoral rocky bottoms and *Posidonia oceanica* beds [27], is a major predator of sea urchins in the Mediterranean [28]; despite its importance, relatively little quantitative information is available on its demography [29]. Understanding the influence of density-dependent processes on its population dynamics, along with the interaction between density dependence and the shelter provided by MPAs, is crucial to assess the effectiveness of the 'reserve effect' in infra-littoral rocky habitats.

### Study site

**Fig 1:**
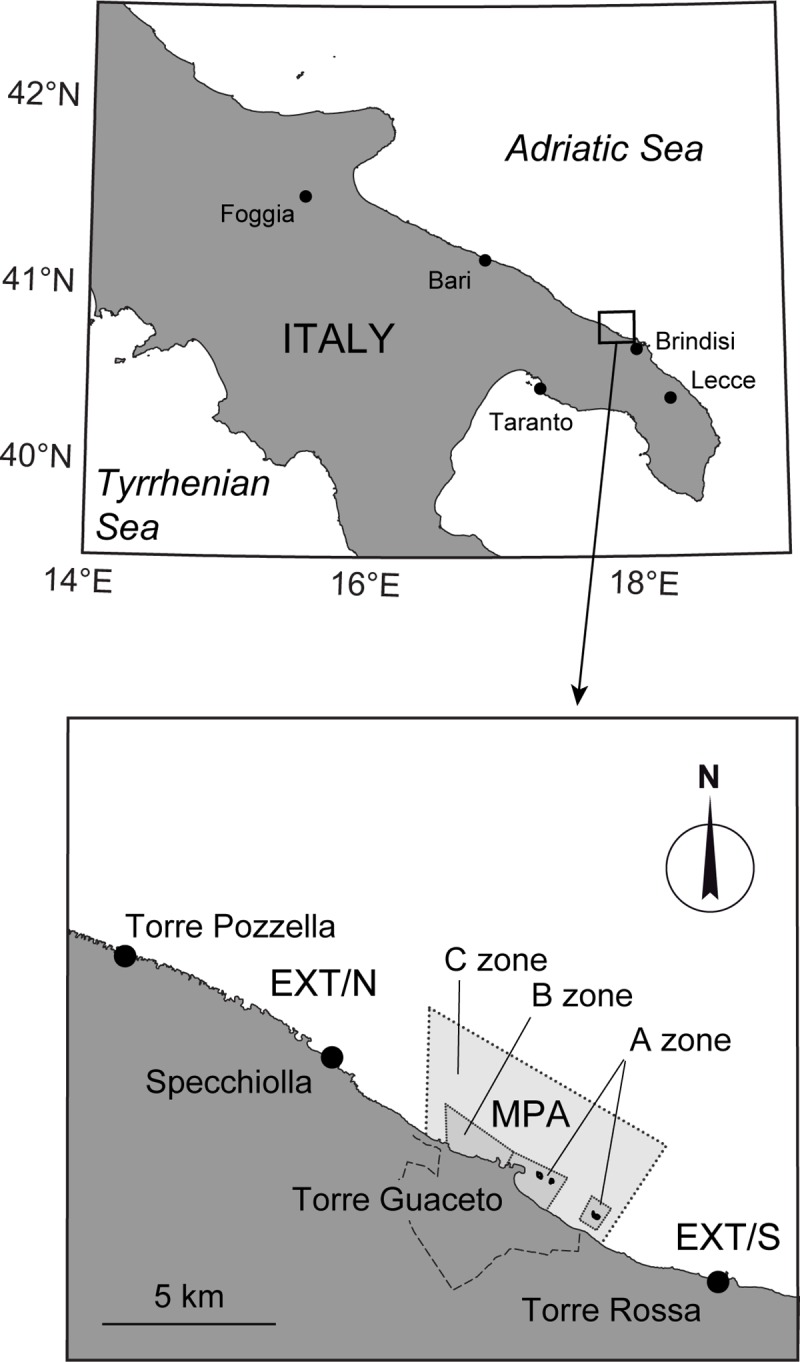
Study area. Torre Guaceto marine protected area (light grey) and sampling sites at unprotected locations (EXT/N and EXT/S). Map based on public domain shapefiles released by the Italian National Institute of Statistics (www.istat.it).

The study area (Fig 1) encompasses a coastline stretch of ~30 km north of Brindisi (Apulia, southern Italy). The whole area is characterized by a rocky plateau gently declining from the surface to a depth of ~10–12 m over coarse sand and *Posidonia oceanica* beds. Coralligenous formations develop at deeper stands (>25–30 m). Torre Guaceto MPA was formally established in 1991, but enforcement became effective around 2000–2001. The MPA covers 2,227 ha and is subdivided into three zones: (1) a no-take reserve (A zone, according to Italian law), where access is restricted to scientists, reserve personnel and police authorities; (2) a general reserve (B zone), where only bathing is allowed; and (3) a partial reserve (C zone), where small scale professional fishing is allowed but restricted to stands deeper than 10 m and to a small number of vessels (≤8) authorized to fish once per week according to a co-management scheme [30]. Recreational fishing is also allowed within the C zone, but only from mid-September to mid-May and under strict regulation. Spearfishing and longlines are totally banned. Torre Guaceto MPA is effectively enforced and provides both conservation and fisheries benefits [31,32]. Outside the protected area, fishing regulations are less restrictive compared to inside, and follow the national Italian law. The whole protected area provides a range of protection for seabreams that can be effectively contrasted against external locations subjected to fishing.

Before the establishment of the MPA, the area was intensively fished. Small-scale professional fishers were quite few (2–3 boats per day were fishing on average in the area) and mostly used trammel nets, while the use of longlines and traps was occasional. Recreational fishers, conversely, were much more numerous and used many techniques, including spearfishing. The most severe fishing impact was, however, the one produced by illegal fishers using explosives, poisons or other toxic chemicals [33]. All of these fishing techniques, illegal or legal, potentially impact *D*. *sargus sargus* populations. Another quantitative information available is that trammel catches per unit effort in the area presently included in the MPA were, before the MPA establishment, similar to the values obtained from the fishing grounds outside the MPA some years after its creation [30,33].

### Data collection

Field sampling was carried out within the no-take zone of Torre Guaceto MPA and at two unprotected locations (Fig 1): one north of the MPA borders (in the following indicated as EXT/N), and another south of the MPA (EXT/S in the following). Unprotected locations were selected at ~4–10 km from the MPA borders and are comparable to protected locations in terms of habitat type, depth, substrate complexity, slope and wave exposure. The distance between protected and unprotected locations is large enough to exclude significant spillover of adult fish [11], but relatively small compared with the dispersal range of propagules (eggs and larvae), which can be transported by currents up to 200 km from spawning areas [34–36], and juveniles, which can disperse over tens of km [35].

Fish density was assessed through underwater visual census (UVC) conducted every year between 2004 and 2015. The research permits for carrying out UVC were provided by the management body of the MPA (Consorzio di Gestione di Torre Guaceto). Censuses were carried out once or twice a year in the central months of the year (from late spring to late summer), concurrently in the protected and unprotected locations to ensure maximum comparability of environmental conditions. Sampling was always performed by the same experienced operators in order to avoid sampling artifacts. Fish abundance and size were recorded along strip transects of 25×5 m, run in ca. 8 min. Locations were randomly selected at stretches of coast characterized by rocky substrate, where other substrate types (such as sand or seagrass) represented less than 5% in cover (both within and around transects), at approximately 5–8 m depth. Only individuals >8 cm TL were counted, because juveniles (i.e. settlers and recruits) do not share the same habitat and depth range of adults. In addition, excluding juveniles from the count avoids the introduction of significant amounts of noise related to the erratic dynamics to which recruitment is subject. Transects were replicated (8 to 32 times per location depending on the year, for a total of 716 replicates) to evaluate uncertainty due to census error and spatial variability at the local scale. Replicates were done at a minimum distance of 50 m from each other to avoid spatial cross-correlation of density estimates. Data on fish density per year and location are reported in (S1 Table).

### Model selection

To assess the role of protection and fish density on the population dynamics of *D*. *sargus sargus*, we carried out a model selection procedure [37] in which we contrasted alternative population dynamics models. The model selection was restricted to unstructured models, because considering also age- or size-structured models would have remarkably increased the number of candidate models to be compared from a little more than 300 (see below) to several thousands. In particular, we considered here the five functional forms used by Brook and Bradshaw [38] in their analysis of density dependence in time series from over 1000 species: two density-independent models (random walk and exponential growth) and three density-dependent models (Ricker-logistic, Gompertz-logistic, and theta-logistic). Since the three locations are open to propagule dispersal and juvenile movement [34–36], we considered an additive error term (instead of a multiplicative on, as in [38]), a choice we deemed more appropriate in this case to represent the effect of possible exchanges of individuals across the region. Therefore, we considered the following relationships:

$$N_{t}{}_{+1}=N_{t}+\varepsilon_{t}\;\left(random\;walk\right)$$ (1A)

$$N_{t+1}=\lambda N_{t}+\varepsilon_{t}\;(\mathrm{exponential}\;\mathrm{growth})$$ (1B)

$$N_{t+1}=\lambda N_{t}\exp\left(-\beta N_{t}\right)+\varepsilon_{t}\;(\mathrm{Ricker}‐\mathrm{logistic})$$ (1C)

$$N_{t+1}=\lambda N_{t}\exp\left(-\beta \log N_{t}\right)+\varepsilon_{t}\;(\mathrm{Gompertz}‐\mathrm{logistic})$$ (1D)

$$N_{t+1}=\lambda N_{t}\exp\left(-\beta N_{t}^{\theta }\right)+\varepsilon_{t}\;(\mathrm{theta}‐\mathrm{logistic})$$ (1E)

where *N*_*t*_ and *N*_*t*+1_ are population densities in year *t* and *t*+1, respectively, *λ* represents the deterministic, density-independent finite growth rate, *β* measures the strength of density dependence, *θ* is an exponent permitting a nonlinear scaling of density dependence, and *ε*_*t*_ is a Gaussian error term with mean 0 and variance *σ*_*ε*_^2^.

To test for possibly different population dynamics among locations, we considered 1) all possible combinations of models (1a–1e) for the three locations separately, 2) models in which the dynamics at two or more locations was described with the same functional form and the same parameters, and 3) models in which one or more locations shared the same functional form and some parameters, but not the others. For instance, a model describing exponential growth at site 1 and Gompertz-logistic dynamics at sites 2 and 3 with the same scaling parameter *β* but different values of the finite growth rate *λ* would be written as follows:

$$N_{1,t+1}=\lambda_{1}N_{1,t}+\varepsilon_{t}$$

$$N_{2,t+1}=\lambda_{2}N_{2,t}\exp\left(-\beta \log N_{t}\right)+\varepsilon_{t}$$

$$N_{3,t+1}=\lambda_{3}N_{3,t}\exp\left(-\beta \log N_{t}\right)+\varepsilon_{t}$$

Overall, we calibrated 337 models (see S2 Table). Then, we looked for the model providing the best compromise between goodness-of-fit and parsimony through a standard model selection procedure based upon the Akaike Information Criterion corrected for finite sample size (AICc [37]).

To avoid possible bias due to census error, instead of fitting the model by regressing the intrinsic population growth rate ln(*N*_*t*+1_/*N*_*t*_) against population density *N*_*t*_, we calibrated it via nonlinear maximum-likelihood estimation (see [39]). For each transition from time *t* to time *t*+1,

- we calculated the prediction error at site *i* as $\varepsilon_{i,t}=N_{i,t+1}-{\hat{N}}_{i,t+1}$, where ${\hat{N}}_{i,t+1}=f(N_{i,t})$ is the estimate of *N*_*i*,*t*+1_ obtained with the specific model (Eqs 1A–1E) to be calibrated;
- we evaluated the Gaussian probability density function at *ε*_*i*,*t*_ (given the error variance *σ*_*ε*_^2^, to be estimated as well) to obtain the likelihood *P*_*t*_ of the transition *N*_*i*,*t*_ → *N*_*i*,*t*+1_;
- we calculated the objective (log-likelihood) function *L* = −∑_*i,t*_ln(*P_i,t_*);
- we found, via numerical optimization, the parameter values that minimize *L*.

In addition, to take properly into account uncertainty associated with both census error and local-scale spatial variability, we adopted a stratified bootstrapping procedure (see [40]) in which we considered each population density estimate from a single transect as the basic data unit. More precisely, after stratifying the data set by year and location,

1. for each year and location, we resampled (with replacement) the transects censused in that year and location (this means that, in a given iteration, some transects might be selected more than once, and some others might not be selected at all);
2. we thus generated a synthetic sample that was used to calibrate the model and derive a new set of parameter estimates;
3. we repeated the two previous steps 1000 times to obtain an empirical probability distribution for each model parameter.

As a measure of central tendency for each parameter we used the median of the corresponding empirical distribution (instead of the mean, as usually done in most bootstrap applications), because of its lower sensitivity to the extreme values that can be generated by bootstrapping [40].

Finally, AIC_c_ weights were calculated to assess the relative strength of evidence of each model, i.e. its likelihood. The Akaike weight *w*_*i*_ of model *i* from a set of *N* models is calculated as

$$w_{i}=\frac{\exp\left(-\frac{1}{2}\Delta_{i}\right)}{\sum_{k=1}^{N}\exp\left(-\frac{1}{2}\Delta_{k}\right)}$$

where Δ_*i*_ is the difference between the AIC_c_ of model *i* and that of the best model (i.e. the one with the minimum AIC_c_), given by Δ_*i*_ = AIC_c *i*_−AIC_c min_ [37]. Akaike weights were used also to evaluate the relative strength of evidence of specific groups of models: in particular, we calculated the overall evidence for density dependence (calculated as the sum of the weights associated to the density-dependent models, see [38]), and the evidence for the hypotheses that 1) all (or some) locations share the same population dynamics, i.e. they share both model structure and parameter values, or 2) that they share the same scaling of density dependence, i.e. the model structure and the value of parameter *β* but not necessarily that of *λ*.

### Population viability analysis

To test whether protection has a significant effect on population viability, we used the model selected through the procedure described above to forecast the fate of the population in the long run. Our approach is similar to that of classical Population Viability Analysis (PVA [41]). Usually, PVA aims to estimate the probability that a population goes extinct or, more generally, falls below a reference threshold (the so-called quasi-extinction threshold [42]) over a fixed time horizon. In our case, however, the population is not closed to immigration from the surroundings and might therefore recover even after a local collapse [43]. For this reason, we indicate this event as a "depletion" rather than a true extinction; nevertheless, a high probability of depletion would provide strong evidence of local overexploitation. We ran Monte Carlo simulations simulating the fate of the three populations over 100 years. To this end, we set the initial condition of each population to the average density observed at its location. Then, we stepped it forward according to the recursive equation given by the corresponding demographic model (Eqs 1A–1E). At each time step, we extracted a value of the error term from a Gaussian distribution with mean zero and standard deviation *σ*_*ε*_ (as obtained via the log-likelihood calibration procedure). We ran a total of 10^5^ simulations, obtaining a set of 100-yr-long synthetic time series for each population. Eventually, we assessed the probability of depletion as the proportion of simulations in which the density of a population fell at least once below the depletion threshold, and the corresponding expected time to depletion as the average time at which the first depletion event occurred.

## Results

**Fig 2:**
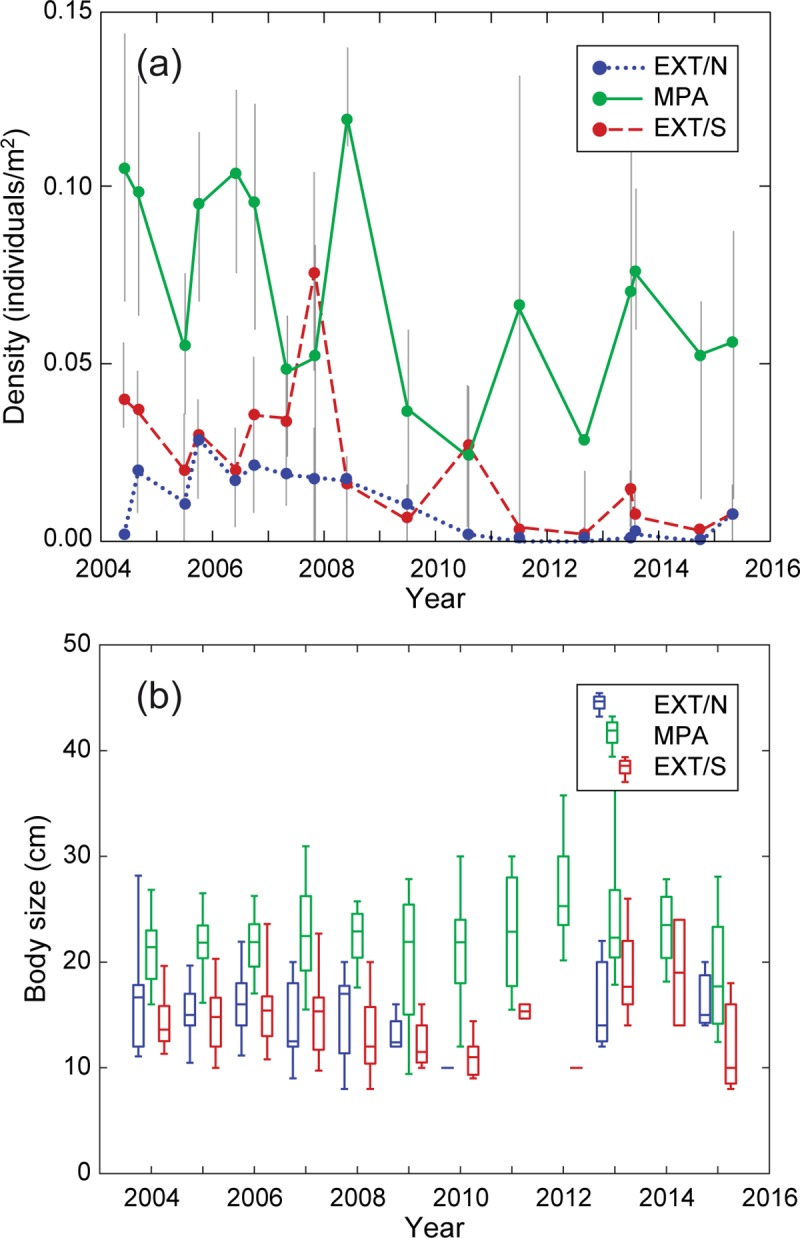
Population density and body size of *Diplodus sargus sargus* inside and outside Torre Guaceto marine protected area. Population density (a) and body size (b) were measured inside the protected area (MPA) and at two unprotected locations outside (EXT/N and EXT/S) via underwater visual census between 2004 and 2015. In (a), dots and vertical bars show mean and interquartile range, respectively, deriving from data variability among transects at the same location. In (b), boxes show the interquartile range (with the horizontal crossing line indicating the median) and whiskers encompass 90% CI.

Data analysis reveals that population abundance (Fig 2A) within the MPA underwent wide fluctuations during the study period, spanning almost an order of magnitude, with average densities ranging between 0.02 and 0.12 individuals/m^2^ (mean ± SD = 0.070 ± 0.029 ind./m^2^). Outside the protected area, seabream density was significantly lower than inside (Kruskal-Wallis test with protection as a factor, χ^2^ = 245.7, *P* < 0.001), with maximum densities below 0.03 individuals/m^2^ (mean ± SD = 0.010 ± 0.009 ind./m^2^) at EXT/N and below 0.08 individuals/m^2^ (mean ± SD = 0.022 ± 0.019 ind./m^2^) at EXT/S. Inter-annual variability was proportionally higher outside the MPA (the coefficient of variation over time of the mean density across transects was 0.92 at EXT/N and 0.96 at EXT/S, respectively) than inside (CV = 0.42). Inside the protected area, seabream size (Fig 2B) was significantly higher than outside (Kruskal-Wallis test on total body length with protection as a factor, χ^2^ = 243.8, *P* < 0.001). Mean (± SD) body length was 21.98 ± 4.32 cm inside, while it was 15.07 ± 3.74 cm outside (with no significant difference between EXT/N and EXT/S; Kruskal-Wallis test with location as a factor, χ^2^ = 3.04, *P* = 0.08).

**Fig 3:**
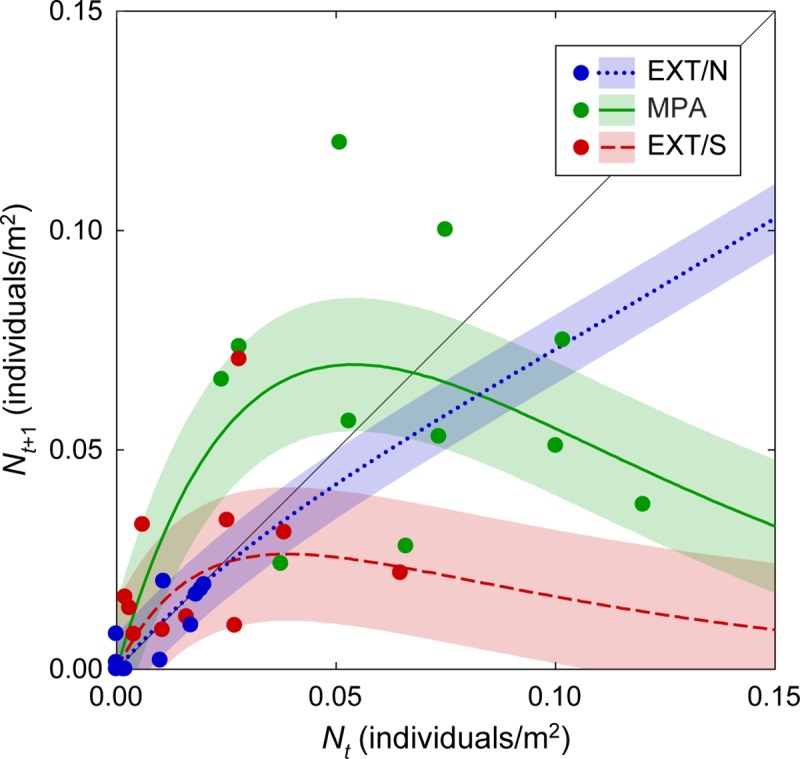
Relationship between population density of *Diplodus sargus sargus* in two subsequent years. Dots indicate data collected inside the protected area (MPA) and at two unprotected locations outside (EXT/N and EXT/S); colored lines and shaded areas are median and interquartile range, respectively, of multi-model predictions (weighted mean of the predictions obtained with the three best models; see text for details). Intersections between the 45° identity line and the colored lines identify equilibrium densities for the deterministic component of the model.

According to the results of the model selection, summarized in Table 1, the best model (i.e. that with the lowest AIC_c_ score) consists of a random-walk dynamics (Eq 1A) for the population at EXT/N, and a Ricker-logistic demography (Eq 1C) for the populations inside the MPA and at EXT/S (see Table 1 for a summary). The dynamics at those two locations share the same parameter for density dependence (*β* = 19.11), but have two different values of the finite growth rate *λ*, much larger for the protected location (*λ*_MPA_ = 3.66) than for the southern location (*λ*_EXT/S_ = 1.49). Two alternative models, however, had performances close to those of the best one: a model (with a ΔAIC_c_ = 0.071) based on a Ricker-logistic relationship with the same *β* for all locations and two different values of *λ* for inside and outside the MPA; and a model (ΔAIC_c_ = 0.324) very similar in structure to the best one, i.e. a random walk for EXT/N and a Ricker-logistic dynamics for both the MPA and EXT/S, but with the same value of *λ* and different values of *β* for MPA and EXT/S, respectively. Subsequent models in the AIC_c_ ranking were separated from the first three by a clear ΔAIC_c_ gap (see S1 Fig), and can thus be considered as sub-optimal. Since there was more than one model with very good performances, we decided to describe and forecast seabream dynamics inside and outside the MPA via multi-model inference [37]: predictions produced by the three best models were combined through a weighted averaging based on AIC_c_ weights. The fitting performances of the resulting model are shown in Fig 3.

**Table 1:** Main statistics of model parameters. Reported values, obtained via bootstrapping, refer to the best model describing the population dynamics of *Diplodus sargus sargus* inside Torre Guaceto MPA and at two unprotected locations (EXT/N and EXT/S). Model residuals were normally distributed (Lilliefors test, *P* > 0.2 for both sub-models).

	*σ*_*ε *EXT/N_	*λ*_MPA_	*λ*_EXT/S_	*β*_MPA-EXT/S_	*σ*_*ε *MPA-EXT/S_
Mean ± SD	0.007 ± 0.002	3.83 ± 1.07	1.50 ± 0.36	19.03 ± 4.22	0.024 ± 0.003
Median (IQR)	0.007 (0.002)	3.66 (1.40)	1.49 (0.40)	19.11 (5.05)	0.024 (0.003)
90% CI	0.005; 0.011	2.51; 5.70	1.09; 2.03	13.40; 25.00	0.020; 0.028

Model settings including theta-logistic relationships had very low support (ΔAIC_c_ > 7). In fact, despite its flexibility the reliability of the theta-logistic model for modelling census data has been questioned due to the trade-off between parameters *β* and *θ* that makes model fitting non-robust. Therefore, in the following of our analysis we left out the theta-logistic when assessing strength of evidence for the different model structures; this choice has the additional advantage of keeping the number of models with and without density dependence balanced. Table 2 shows the relative strength of evidence for the different population dynamics, i.e. the sum of the Akaike weights of all the models belonging to a given family. The most supported model form was the Ricker-logistic both inside the MPA (49%) and at EXT/S (42%), while it was a random walk at EXT/N (63%). The overall strength of evidence for density dependence was equal to 83% inside the MPA, 66% and 11% outside (EXT/S and EXT/N, respectively). There was no evidence (<1%) that the same type of population dynamics occurs in all locations, nor that EXT/N shares the same dynamics with either EXT/S or the MPA (1% and <1%, respectively). In contrast, there was some evidence (27%) that the same demographic mechanism drives population dynamics in the MPA and at EXT/S.

**Table 2:** Relative strength of evidence for five model variants. Model variants describe the population dynamics of *Diplodus sargus sargus* inside Torre Guaceto MPA and at two unprotected locations (EXT/N and EXT/S) with alternative formulations (see Eqs 1A–1E). Strength of evidence is calculated as the sum of the Akaike weights of the models belonging to a given model variant: for instance, the value of 8.2 reported in the first row of the first column of the table indicates that the sum of the Akaike weights of the 47 models in which the dynamics inside the MPA is described as a random walk is equal to 8.2%.

	MPA	EXT/N	EXT/S
*Density-independent models*			
Random walk	8.2	62.8	13.9
Exponential growth	8.9	26.0	20.5
*Density-dependent models*			
Ricker-logistic growth	49.4	5.8	41.9
Gompertz-logistic growth	33.5	5.4	23.7

**Fig 4:**
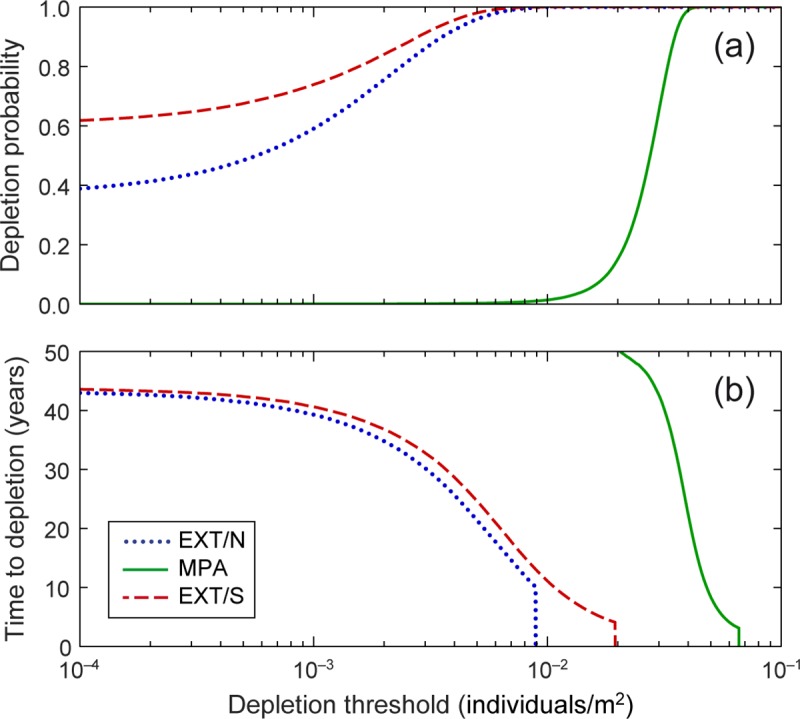
Results of the Population Viability Analysis inside and outside Torre Guaceto marine protected area. Depletion probability (a) and expected time to depletion (b) inside the protected area (MPA) and at two unprotected locations outside (EXT/N and EXT/S) are shown as functions of the depletion threshold and were estimated via Monte Carlo simulation (10^5^ iterations) and multi-model inference (see text for details).

The results of the population viability analysis show that, inside the MPA, the probability of depletion within a 100-year horizon (Fig 4A) is negligible for values of the depletion threshold up to 0.01 individuals/m^2^ (the order of magnitude of the minimum observed density). On the other hand, depletion outside the MPA is quite likely (ca. 40% and 60% at EXT/N and EXT/S, respectively) even for values of the threshold as low as 10^−4^ individuals/m^2^ (about 1/100 of the average density observed in unprotected locations). For this value of the threshold, the expected time to depletion outside the protected area (Fig 4B) is around 45 years, decreasing to less than 40 years when the threshold is set to 10^−3^ individuals/m^2^ (1/10 of the average density). Note that, since the expected time to depletion is calculated only on those simulations that lead to a depletion event, its value becomes barely significant when the probability of depletion is very low. This is the case, in particular, for the population inside the MPA, where for thresholds values <0.01 individuals/m^2^ the probability of depletion is below 2% and time to depletion is therefore calculated only on a small number of simulations. As the threshold increases, time to depletion decreases at each location, and approaches zero as the threshold attains the average population density observed at that location, i.e. the initial condition of the corresponding simulations. Beyond this limit, time to depletion equals zero by definition (because population density is below the threshold from the beginning).

## Discussion

We developed a modeling framework based on nonparametric statistics, model selection and multi-model inference to contrast alternative hypotheses about how density dependence affects demographic dynamics under protected and unprotected conditions. Although the proposed approach builds upon well-established techniques, it is novel in several aspects. Bootstrap resampling, by explicitly taking into account the uncertainty that affects data collected in a fluctuating and spatially heterogeneous environment, allows for the empirical integration of census error into the analysis. The model selection procedure provides an indirect (comparative) way to assess the relevance of density dependence in regulating population dynamics. This avoids both the spurious detection of density dependence (as in tests based on correlative methods) and the need for *a priori* assumptions on the mechanisms linking population density and population growth rate. On the contrary, alternative hypotheses can be contrasted in terms of evidence strength by using Akaike weights produced via the model selection procedure, which can also be used to assess whether specific dynamics are shared across protected and unprotected populations [37,38].

Population density affects a number of life history traits, including survival, body growth, reproduction, access to resources, and movement (see [3] for an extensive review), and can ultimately affect the success of marine protection [44]. Our results suggest that population density plays a significant role in regulating the population size of *Diplodus sargus sargus* inside Torre Guaceto MPA. The output of the model selection supports the view that density dependence follows an overcompensative mechanism. Overcompensation (a type of density dependence in which recruitment not only levels off, but eventually decreases when population density exceeds a threshold [45]), like that described by the Ricker-logistic model, can arise from competition for resources taking the form of a scramble (in which all individuals divide the resources equally) instead of a contest, in which only the strongest individuals gain access to the resources, generally determining a simply compensatory dynamics [46].

In our study, overall evidence for density dependence was much lower at the unprotected location north of the MPA (EXT/N) than inside Torre Guaceto MPA and the unprotected location south of it (EXT/S). A possible interpretation for this result is that the data collected at EXT/N span only a small range of variation compared with the other two locations: consequently, the range of observed fish densities may be too narrow to provide sufficiently robust evidence about the actual demographic dynamics at that specific location. In fact, the structure of the second best model indicates that population dynamics at the two unprotected locations (EXT/N and EXT/S) are also regulated by population density and cannot be distinguished from each other. If population dynamics at the two unprotected locations were actually different (as in the first and third best models), this might be justified by a differential recruitment subsidy provided by the MPA after every annual reproduction event, i.e. around April-June. Recent studies report that *Diplodus sargus sargus* commonly spawns between 0 and 80 m depth [47,48]. With specific reference to Torre Guaceto, previous studies have suggested that spawning events could take place within and close to the protected area [49–51], but information at higher spatial resolution is not available. Wherever the spawning events take place in the south-western Adriatic Sea (where the studied MPA is located), one important point to take into account is that circulation patterns along Apulian coasts are dominated by the Western Adriatic Coastal Current, which most likely originates a large and persistent export of propagules from the MPA toward south. Previous studies on long-distance dispersal of white seabream larvae [34–36], as well as recent studies on seascape connectivity in the Adriatic basin [52–54], strongly support this interpretation and provide a plausible explanation of the similarity between the demographic dynamics observed inside the MPA and at the southern unprotected location. The patterns observed in terms of different population densities north and south of the MPA could reflect into different spillover rates across the northern or southern MPA boundaries.

In our model, we considered a stochastic term that can account for the possible exchange of individuals across the borders of the populations under study. However, we did not consider explicitly the exchange of individuals between the three locations, since the distance between protected and unprotected locations was deemed large enough to exclude a significant adult spillover (see [11] and references therein) but relatively small compared with the range of propagule and juvenile dispersal [35,55,56]. High densities of large fish, such as those attained within the MPA, can not only enhance recovery from recruitment fluctuations thanks to the storage effect [57], but also provide substantial recruitment subsidy [58]. A more comprehensive modelling approach, taking into account the effect of recruitment subsidy from a location to another, and in particular from the MPA to its surroundings, would help cast light on how local dynamics are interconnected by fish dispersal. To this end, however, information on local demographic dynamics should be properly integrated into a wider metapopulation model.

Differences in body size and population growth rate between protected and unprotected conditions quantitatively confirm that the MPA provides effective protection to the local white seabream population, as already shown by previous work [25,34,59]. If differences in population growth rates could be completely ascribed to the fishing pressure exerted outside the MPA, an upper bound estimate of fishing mortality rate *F* could be obtained from the ratio between population growth rate inside and outside the MPA. In fact, if λ_MPA_ = exp(–*M*), where *M* is the natural mortality rate (assumed equal inside and outside the MPA), and λ_EXT_ = exp(–*Z*), where *Z* = *M* + *F* is the total mortality rate outside the MPA, then the fishing mortality rate can be calculated as *F* = ln(λ_MPA_/ λ_EXT_). This yields a value of *F* = 0.56 year^–1^ at EXT/S and 1.13 year^–1^ at EXT/N, roughly indicating that fishing may remove up to 43% and 68% of the population (at EXT/S and EXT/N, respectively) each year. These values are higher than those reported in the literature for other sparid populations: Pajuelo and Lorenzo [60] indicate *F* = 0.37 year^–1^ for *D*. *sargus cadenati* off Canary Islands; Froese et al. [61] report the same value for *D*. *sargus sargus* in the Aegean Sea, while Mahmud et al. [62] report *F* = 0.49 year^–1^ for *D*. *sargus sargus* in Abu Qir Bay, Egypt. The discrepancy between our estimate and the others may be partly explained by the different approaches used to estimate *F*; most likely, however, it has to be ascribed in large part to the remarkable difference of population growth rates between protected and unprotected conditions. The high value of *λ* inside the MPA can be explained by enhanced recruitment due to the presence of large spawners; on the other hand, low population growth rates outside the MPA are likely determined by the combined effect of net migratory balance and fishing pressure.

As for the first factor (net migratory balance), the study area is actually characterized by high seascape connectivity, without appreciable differences in retention and source/sink strength [63]. Local circulation patterns, characterized by a southward current flow, suggest that the export of propagules from Torre Guaceto benefits sites located south rather than north of the MPA, possibly explaining the difference between the two unprotected locations. On the other hand, the second factor (fishing pressure) may explain the residual discrepancy in population growth rates between the southern unprotected location and the interior of the MPA. Although for Torre Guaceto there are no available assessments of population density before the establishment of the MPA, and despite the lack of reliable data about the actual fishing effort in its surroundings, it is well known that fishing effort in the region is rather high [64], and that illegal fishing is also practiced outside the MPA. Other factors, such as environmental features (other than water circulation) or predation are unlikely to have a differential effect on population growth rate across the study area [65]. In fact, environmental features are fairly homogeneous across the three locations. Predation would likely determine larger impacts on small to medium-sized consumers, such as *D*. *sargus sargus*, in the interior of the MPA, where population density is higher, than at the unprotected locations [66], so its effect would eventually mitigate, rather than exacerbate, differences in population growth rate.

A striking effect of protection on the long-term population dynamics of *D*. *sargus sargus* emerges from the results of the population viability analysis. While population depletion within the protected area appears very unlikely, the depletion probability for the two unprotected populations is, in contrast, very high. This is not surprising for the population at the northern location, whose dynamics appear quite erratic and may thus be driven more by the exchange of individuals with the surroundings than by local demographic mechanisms. Conversely, the fact that the estimated probability of depletion at the southern location is higher than that at the northern one can be surprising at first, especially in the light of the directional recruitment subsidy provided by Torre Guaceto MPA to unprotected locations discussed above. If demographic dynamics represented by our models were purely deterministic, one would indeed expect each population to tend toward an equilibrium density, determined by the intersection between the solid curves in Fig 3 and the 45° identity line [67]. Population density at equilibrium would then be approximately three times higher in the MPA (0.068 individuals/m^2^) than at EXT/S (0.024 ind./m^2^), and even higher compared to that at EXT/N (0.018 ind./m^2^). In the stochastic version of the model, the probability of depletion is determined by the interaction between the mechanistic component of demographic dynamics and the effect of environmental noise. The population at EXT/S is characterized by both stronger overcompensation and a larger noise term than that at EXT/N, and their combined effect can cause a depletion event not only when population density is low, but also when it is particularly high. Since the noise term accounts also for possible exchanges of individuals across the region, the wider variation to which the population at the southern location is subject may be caused by wider fluctuations in demographic rates, more intense and variable exchanges with the surroundings, or a combination of the two factors (see e.g. [68]).

## Conclusion

In conclusion, our results strongly support previous evidence that Torre Guaceto MPA provides effective protection to the local white seabream population. Population density within the MPA seems to vary, albeit with remarkable fluctuations, around a value determined by density-dependent mechanisms. To what extent these fluctuations reflect a true demographic process and to what they are the consequence of census error it is difficult to say: in any case, the uncertainty affecting our knowledge about the actual population size should be taken into account when developing management strategies for the MPA and its surroundings.

Even if density dependence sets a limit on the maximum population size that can be hosted within the MPA, density-dependent dispersal can extend the benefits of protection towards unprotected areas surrounding the MPA. Benefits, in terms of recruitment subsidy, can extend also to farther locations as long as they are supplied with juveniles through the ocean circulation system. While the establishment and effective management of MPAs is crucial to support the preservation of viable fish populations within their borders, the conservation of marine living resources at larger scales requires appropriate design and enforcing of fisheries management policies informed by sound ecological knowledge. A realistic description of demographic dynamics, including density dependent mechanisms, is key to designing effective systems of MPAs [69]. Sustainable fisheries management calls for an inclusive approach taking explicitly into account the environmental, biological and socioeconomic factors at play and their complex interaction through time and space [70]. From the institutional side this requires a more effective assessment and control of illegal fishing, as well as the active involvement of stakeholders in the planning and decision process to identify major issues related to the establishment of MPAs, promote reciprocal understanding, and encourage the generation of new options and solutions [35,71].

## Supporting information

S1 Fig AICc ranking of the best 20 demographic models. (TIF)

S1 Table Density of *Diplodus sargus sargus* inside and outside Torre Guaceto marine protected area. (DOCX)

S2 Table Full list of models considered in the analysis. (DOCX)
