# Supplementary figures and images for: Protection reveals density-dependent dynamics in fish populations: A case study in the central Mediterranean
Source: PLoS One. 2020 Feb 3;15(2):e0228604. doi: 10.1371/journal.pone.0228604 (PMC6996820; doi:10.1371/journal.pone.0228604)

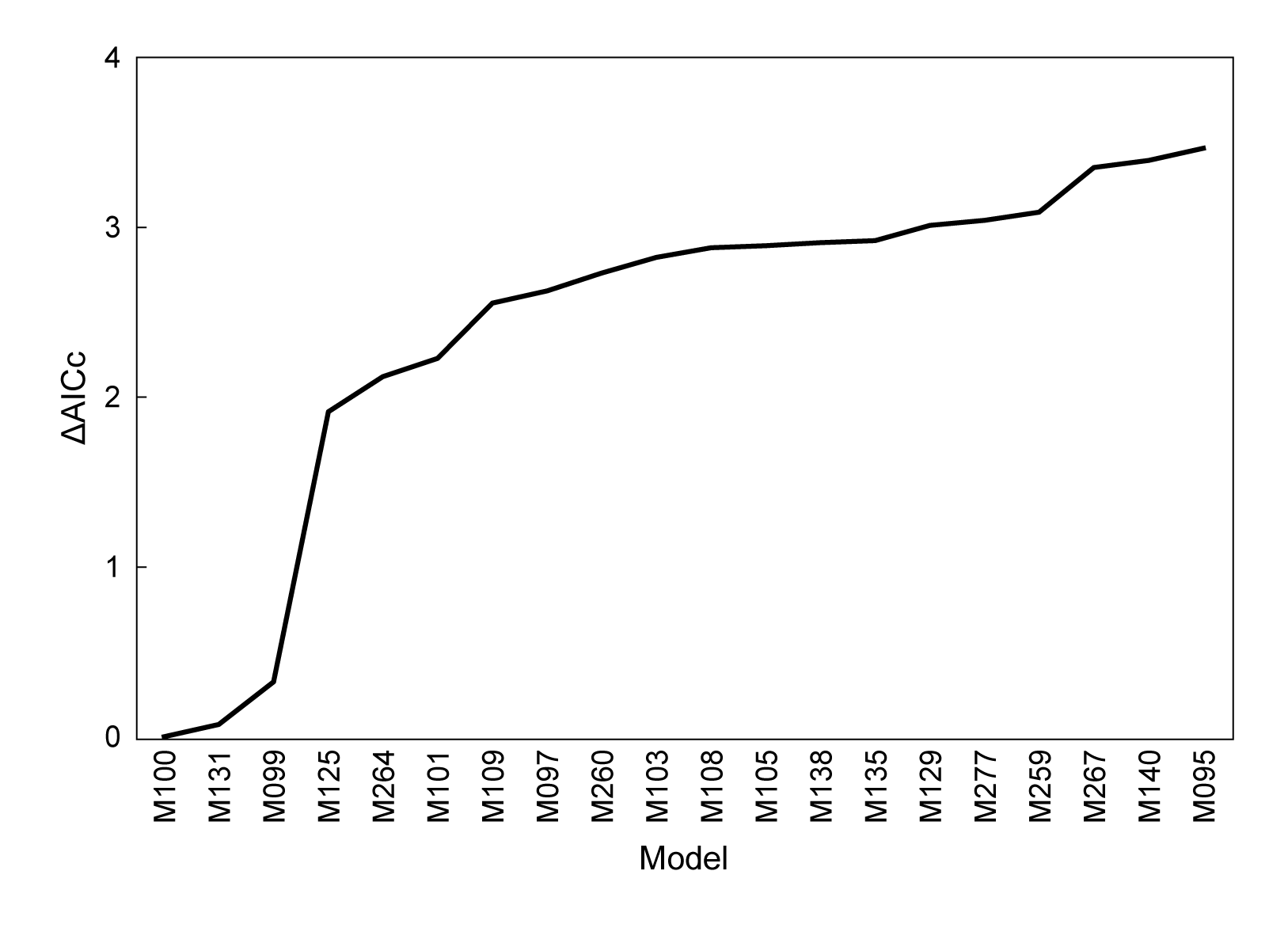

Supplement: S1 Fig — (TIF) [file pone.0228604.s001.tif]
