# Supplementary material for: Protection reveals density-dependent dynamics in fish populations: A case study in the central Mediterranean
Source: PLoS One. 2020 Feb 3;15(2):e0228604. doi: 10.1371/journal.pone.0228604 (PMC6996820; doi:10.1371/journal.pone.0228604)
Supplement: S1 Table — (DOCX) [file pone.0228604.s002.docx]

**Table S1. Density of *Diplodus sargus sargus* inside and outside Torre Guaceto marine protected area.** For each year and location, mean and standard deviation (SD) of fish density (individuals/m^2^) inside the protected area (MPA) and at two unprotected locations outside (EXT/N and EXT/S) are reported, along with the number of replicates (*n*).

| **Year** | **Location** | **Mean** | **SD** | ***n*** |
| --- | --- | --- | --- | --- |
| 2004 | EXT/N | 0.0108 | 0.0145 | 32 |
| 2004 | EXT/S | 0.0383 | 0.0241 | 32 |
| 2004 | MPA | 0.1018 | 0.0454 | 64 |
| 2005 | EXT/N | 0.0200 | 0.0233 | 32 |
| 2005 | EXT/S | 0.0253 | 0.0214 | 32 |
| 2005 | MPA | 0.0750 | 0.0392 | 64 |
| 2006 | EXT/N | 0.0193 | 0.0164 | 32 |
| 2006 | EXT/S | 0.0280 | 0.0206 | 32 |
| 2006 | MPA | 0.1001 | 0.0380 | 64 |
| 2007 | EXT/N | 0.0182 | 0.0248 | 11 |
| 2007 | EXT/S | 0.0647 | 0.0494 | 11 |
| 2007 | MPA | 0.0509 | 0.0545 | 22 |
| 2008 | EXT/N | 0.0170 | 0.0220 | 8 |
| 2008 | EXT/S | 0.0160 | 0.0121 | 8 |
| 2008 | MPA | 0.1200 | 0.0268 | 16 |
| 2009 | EXT/N | 0.0100 | 0.0140 | 8 |
| 2009 | EXT/S | 0.0060 | 0.0071 | 8 |
| 2009 | MPA | 0.0375 | 0.0312 | 16 |
| 2010 | EXT/N | 0.0020 | 0.0057 | 8 |
| 2010 | EXT/S | 0.0270 | 0.0296 | 8 |
| 2010 | MPA | 0.0240 | 0.0327 | 16 |
| 2011 | EXT/N | 0.0000 | 0.0000 | 8 |
| 2011 | EXT/S | 0.0040 | 0.0086 | 8 |
| 2011 | MPA | 0.0660 | 0.0954 | 16 |
| 2012 | EXT/N | 0.0000 | 0.0000 | 8 |
| 2012 | EXT/S | 0.0020 | 0.0037 | 8 |
| 2012 | MPA | 0.0280 | 0.0500 | 16 |
| 2013 | EXT/N | 0.0015 | 0.0032 | 16 |
| 2013 | EXT/S | 0.0105 | 0.0126 | 16 |
| 2013 | MPA | 0.0735 | 0.0620 | 32 |
| 2014 | EXT/N | 0.0000 | 0.0000 | 8 |
| 2014 | EXT/S | 0.0030 | 0.0056 | 8 |
| 2014 | MPA | 0.0530 | 0.0574 | 16 |
| 2015 | EXT/N | 0.0080 | 0.0142 | 8 |
| 2015 | EXT/S | 0.0080 | 0.0121 | 8 |
| 2015 | MPA | 0.0565 | 0.0520 | 16 |
| 2004 | EXT/N | 0.0108 | 0.0145 | 32 |
