# Supplementary material for: Protection reveals density-dependent dynamics in fish populations: A case study in the central Mediterranean
Source: PLoS One. 2020 Feb 3;15(2):e0228604. doi: 10.1371/journal.pone.0228604 (PMC6996820; doi:10.1371/journal.pone.0228604)
Supplement: S2 Table — (DOCX) [file pone.0228604.s003.docx]

**Table S2. Full list of models considered in the analysis.** For each model, a brief description is provided along with the corresponding values of AICc, ΔAICc and *w* (Akaike weight). Models are sorted by increasing AICc. RW: random walk (eq. 1a in the main text); EX: exponential growth (eq. 1b); RL: Ricker-logistic dynamics (eq. 1c); GL: Gompertz-logistic dynamics (eq. 1d); TL: theta-logistic dynamics (eq. 1e). MPA: protected location inside Torre Guaceto MPA; EXT/N: unprotected location north of the MPA; EXT/S: unprotected location south of the MPA.

| **ID** | **Synthetic model description** | **AICc** | **ΔAICc** | ***w*** |
| --- | --- | --- | --- | --- |
| M100 | RW at EXT/N, RL with same *β* and different *λ* at MPA and EXT/S | –256.0 | 0.0 | 1.0×10^–1^ |
| M131 | RL with same *λ* at EXT/N and EXT/S, different at MPA, same *β* at all locations | –255.9 | 0.1 | 1.0×10^–1^ |
| M099 | RW at EXT/N, RL with same *λ* and different *β* at MPA and EXT/S | –255.7 | 0.3 | 8.8×10^–2^ |
| M125 | RL with same *λ* at all locations, same *β* at EXT/N and EXT/S, different at MPA | –254.1 | 1.9 | 4.0×10^–2^ |
| M264 | RW at EXT/N, GL with same *β* and different *λ* at MPA and EXT/S | –253.9 | 2.1 | 3.6×10^–2^ |
| M101 | RW at EXT/N, RL with different parameters at MPA and EXT/S | –253.8 | 2.2 | 3.4×10^–2^ |
| M109 | EX at EXT/N, RL with same *β* and different *λ* at MPA and EXT/S | –253.5 | 2.6 | 2.9×10^–2^ |
| M097 | RW at EXT/N, EX at EXT/S RL at MPA | –253.4 | 2.6 | 2.8×10^–2^ |
| M260 | RW at EXT/N, RL at EXT/S, GL at MPA | –253.3 | 2.7 | 2.6×10^–2^ |
| M103 | RW at EXT/N, RL at MPA, GL at EXT/S | –253.2 | 2.8 | 2.5×10^–2^ |
| M108 | EX at EXT/N, RL with same *λ* and different *β* at MPA and EXT/S | –253.1 | 2.9 | 2.5×10^–2^ |
| M105 | EX with same *λ* at EXT/N and EXT/S, RL at MPA | –253.1 | 2.9 | 2.4×10^–2^ |
| M138 | RL with same *λ* at EXT/N and EXT/S, different at MPA, same *β* at MPA and EXT/S, different at EXT/N | –253.1 | 2.9 | 2.4×10^–2^ |
| M135 | RL with same *λ* at MPA and EXT/S, different at EXT/N, same *β* at MPA and EXT/N, different at EXT/S | –253.1 | 2.9 | 2.4×10^–2^ |
| M129 | RL with same *λ* at MPA and EXT/N, different at EXT/S, same *β* at MPA and EXT/S, different *β* at EXT/N | –253.0 | 3.0 | 2.3×10^–2^ |
| M277 | RL with same parameters at EXT/N and EXT/S, GL at MPA | –253.0 | 3.0 | 2.3×10^–2^ |
| M259 | RW at EXT/N, EX at EXT/S, GL at MPA | –252.9 | 3.1 | 2.2×10^–2^ |
| M267 | EX with same *λ* at EXT/N and EXT/S, GL at MPA | –252.7 | 3.4 | 1.9×10^–2^ |
| M140 | RL with same *λ* at MPA and EXT/S, different at EXT/N, same *β* at EXT/N and EXT/S, different at MPA | –252.6 | 3.4 | 1.9×10^–2^ |
| M095 | RW at EXT/N and EXT/S with same variance, RL at MPA | –252.5 | 3.5 | 1.8×10^–2^ |
| M096 | RW at EXT/N and EXT/S with different variance, RL at MPA | –252.5 | 3.5 | 1.8×10^–2^ |
| M257 | RW at EXT/N and EXT/S with same variance, GL at MPA | –252.1 | 3.9 | 1.5×10^–2^ |
| M258 | RW at EXT/N and EXT/S with different variance, GL at MPA | –252.1 | 3.9 | 1.5×10^–2^ |
| M128 | RL with same *λ* at MPA and EXT/N, different at EXT/S, same *β* at EXT/N and EXT/S, different *β* at MPA | –251.5 | 4.5 | 1.1×10^–2^ |
| M273 | EX at EXT/N, GL with same *β* and different *λ* at MPA and EXT/S | –251.3 | 4.7 | 1.0×10^–2^ |
| M110 | EX at EXT/N, RL with different parameters at MPA and EXT/S | –251.0 | 5.0 | 8.6×10^–3^ |
| M106 | EX with different *λ* at EXT/N and EXT/S, RL at MPA | –250.8 | 5.2 | 7.8×10^–3^ |
| M119 | EX at EXT/S, RL with same *β* and different *λ* at MPA and EXT/N | –250.8 | 5.2 | 7.7×10^–3^ |
| M169 | RL with same *β* and different *λ* at MPA and EXT/S, GL at EXT/N | –250.6 | 5.4 | 7.0×10^–3^ |
| M269 | EX at EXT/N, RL at EXT/S GL at MPA | –250.5 | 5.5 | 6.7×10^–3^ |
| M172 | RL at MPA, GL with same *λ* and *β* at EXT/N and EXT/S | –250.5 | 5.5 | 6.6×10^–3^ |
| M118 | EX at EXT/S, RL with same *λ* and different *β* at MPA and EXT/N | –250.4 | 5.6 | 6.4×10^–3^ |
| M112 | EX at EXT/N, RL at MPA, GL at EXT/S | –250.4 | 5.6 | 6.4×10^–3^ |
| M152 | RL with same *β* and different *λ* at MPA and EXT/N, GL at EXT/S | –250.4 | 5.6 | 6.3×10^–3^ |
| M268 | EX with different *λ* at EXT/N and EXT/S, GL at MPA | –250.4 | 5.6 | 6.2×10^–3^ |
| M168 | RL with same *λ* and different *β* at MPA and EXT/S, GL at EXT/N | –250.3 | 5.7 | 6.0×10^–3^ |
| M104 | RW at EXT/S, EX at EXT/N, RL at MPA | –250.2 | 5.9 | 5.5×10^–3^ |
| M115 | RW at EXT/N, RL with same *β* and different *λ* at MPA and EXT/N | –250.1 | 5.9 | 5.5×10^–3^ |
| M122 | RL with same *λ* at all locations, same *β* at MPA and EXT/N, different at EXT/S | –250.1 | 5.9 | 5.3×10^–3^ |
| M151 | RL with same *λ* and different *β* at MPA and EXT/N, GL at EXT/S | –250.0 | 6.0 | 5.2×10^–3^ |
| M134 | RL with different *λ* at each location, same *β* at all locations | –250.0 | 6.0 | 5.1×10^–3^ |
| M114 | RW at EXT/N, RL with same *λ* and different *β* at MPA and EXT/N | –249.8 | 6.2 | 4.7×10^–3^ |
| M266 | RW at EXT/S, EX at EXT/N, GL at MPA | –249.7 | 6.3 | 4.5×10^–3^ |
| M127 | RL with same *λ* at all locations, different *β* at each location | –249.6 | 6.4 | 4.3×10^–3^ |
| M123 | RL with same *β* at all locations, same *λ* at MPA and EXT/N, different at EXT/S | –249.4 | 6.6 | 3.7×10^–3^ |
| M319 | GL with same *λ* at all locations, different *β* at each location | –249.2 | 6.8 | 3.4×10^–3^ |
| M278 | RL with same *λ* and different *β* at EXT/N and EXT/S, GL at MPA | –248.7 | 7.3 | 2.7×10^–3^ |
| M284 | RL at EXT/N, GL with same *β* and different *λ* at MPA and EXT/S | –248.5 | 7.5 | 2.5×10^–3^ |
| M098 | RW at EXT/N, RL with same parameters at MPA and EXT/S | –248.3 | 7.7 | 2.2×10^–3^ |
| M174 | RL at MPA, GL with same *β* and different *λ* at EXT/N and EXT/S | –248.0 | 8.0 | 1.9×10^–3^ |
| M166 | EX at EXT/S, RL at MPA, GL at EXT/N | –248.0 | 8.0 | 1.9×10^–3^ |
| M102 | RW at EXT/N, RL at MPA, TL at EXT/S | –248.0 | 8.0 | 1.9×10^–3^ |
| M321 | GL with same *λ* at MPA and EXT/N, different at EXT/S, same *β* at MPA and EXT/S, different at EXT/N | –248.0 | 8.0 | 1.9×10^–3^ |
| M330 | GL with same *λ* at EXT/N and EXT/S, different at MPA, same *β* at MPA and EXT/S, different at EXT/N | –248.0 | 8.0 | 1.9×10^–3^ |
| M170 | RL with different parameters at MPA and EXT/S, GL at EXT/N | –248.0 | 8.0 | 1.9×10^–3^ |
| M120 | EX at EXT/S, RL with different parameters at MPA and EXT/N | –248.0 | 8.0 | 1.9×10^–3^ |
| M145 | RL with different parameters at each location | –247.9 | 8.1 | 1.8×10^–3^ |
| M132 | RL with same *λ* at EXT/N and EXT/S, different at MPA, same *β* at MPA and EXT/N, different at EXT/S | –247.9 | 8.1 | 1.8×10^–3^ |
| M160 | RL at MPA, TL with same parameters at EXT/N and EXT/S | –247.7 | 8.3 | 1.6×10^–3^ |
| M139 | RL with same *λ* at EXT/N and EXT/S, different at MPA, different *β* at each location | –247.7 | 8.3 | 1.6×10^–3^ |
| M158 | RL with same *β* and different *λ* at MPA and EXT/S, TL at EXT/N | –247.6 | 8.4 | 1.6×10^–3^ |
| M117 | EX at EXT/S, RL with same parameters at MPA and EXT/N | –247.6 | 8.4 | 1.5×10^–3^ |
| M276 | EX at EXT/S, RL at EXT/N, GL at MPA | –247.6 | 8.4 | 1.5×10^–3^ |
| M261 | RW at EXT/N, TL at EXT/S, GL at MPA | –247.5 | 8.5 | 1.5×10^–3^ |
| M165 | RW at EXT/S, RL at MPA, GL at EXT/N | –247.5 | 8.5 | 1.5×10^–3^ |
| M116 | RW at EXT/N, RL with different parameters at MPA and EXT/N | –247.5 | 8.5 | 1.5×10^–3^ |
| M280 | RL with different parameters at EXT/N and EXT/S, GL at MPA | –247.5 | 8.5 | 1.5×10^–3^ |
| M153 | RL with different parameters at MPA and EXT/N, GL at EXT/S | –247.4 | 8.6 | 1.4×10^–3^ |
| M157 | RL with same *λ* and different *β* at MPA and EXT/S, TL at EXT/N | –247.3 | 8.7 | 1.3×10^–3^ |
| M289 | TL with same parameters at EXT/N and EXT/S, GL at MPA | –247.3 | 8.7 | 1.3×10^–3^ |
| M113 | RW at EXT/N, RL with same parameters at MPA and EXT/N | –247.2 | 8.8 | 1.3×10^–3^ |
| M175 | RL at MPA, GL with different parameters at EXT/N and EXT/S | –247.2 | 8.8 | 1.3×10^–3^ |
| M275 | RW at EXT/S, RL at EXT/N, GL at MPA | –247.1 | 8.9 | 1.2×10^–3^ |
| M302 | EX at EXT/S, GL with same *λ* and different *β* at MPA and EXT/N | –247.1 | 8.9 | 1.2×10^–3^ |
| M144 | RL with different *λ* at each location, same *β* at MPA and EXT/S, different at EXT/N | –247.1 | 8.9 | 1.2×10^–3^ |
| M150 | RL with same parameters at MPA and EXT/N, GL at EXT/S | –247.0 | 9.0 | 1.1×10^–3^ |
| M306 | RL at EXT/S, GL with same *λ* and different *β* at MPA and EXT/N | –247.0 | 9.0 | 1.1×10^–3^ |
| M279 | RL with same *β* and different *λ* at EXT/N and EXT/S, GL at MPA | –247.0 | 9.0 | 1.1×10^–3^ |
| M143 | RL with same *λ* at MPA and EXT/S, different at EXT/N, different *β* at each location | –246.9 | 9.1 | 1.1×10^–3^ |
| M298 | RW at EXT/S, GL with same *λ* and different *β* at MPA and EXT/N | –246.8 | 9.2 | 1.0×10^–3^ |
| M121 | RL with same parameters at all locations | –246.8 | 9.2 | 1.0×10^–3^ |
| M209 | EX at EXT/S, TL with same parameters at MPA and EXT/N | –246.7 | 9.3 | 1.0×10^–3^ |
| M213 | RL at EXT/S, TL with same parameters at MPA and EXT/N | –246.6 | 9.4 | 9.2×10^–4^ |
| M205 | RW at EXT/S, TL with same parameters at MPA and EXT/N | –246.4 | 9.6 | 8.6×10^–4^ |
| M242 | TL with same parameters at MPA and EXT/N, GL at EXT/S | –246.0 | 10.0 | 7.1×10^–4^ |
| M107 | EX at EXT/N, RL with same parameters at MPA and EXT/S | –245.9 | 10.1 | 6.6×10^–4^ |
| M133 | RL with same *λ* at MPA and EXT/S, different at EXT/N, same *β* at all locations | –245.9 | 10.1 | 6.6×10^–4^ |
| M263 | RW at EXT/N, GL with same *λ* and different *β* at MPA and EXT/S | –245.9 | 10.1 | 6.5×10^–4^ |
| M126 | RL with same *λ* at all locations, same *β* at MPA and EXT/N, different at EXT/S | –245.8 | 10.2 | 6.3×10^–4^ |
| M262 | RW at EXT/N, GL with same *λ* and *β* at MPA and EXT/S | –245.8 | 10.2 | 6.2×10^–4^ |
| M295 | TL at EXT/N, GL with same *β* and different *λ* at MPA and EXT/S | –245.5 | 10.5 | 5.5×10^–4^ |
| M136 | RL with different *λ* at each location, same *β* at MPA and EXT/N, different at EXT/S | –245.5 | 10.5 | 5.4×10^–4^ |
| M336 | GL with different *λ* at each location, same *β* at MPA and EXT/S, different at EXT/N | –245.2 | 10.8 | 4.6×10^–4^ |
| M183 | RW at EXT/N, TL with different parameters at MPA and EXT/S | –245.1 | 10.9 | 4.5×10^–4^ |
| M217 | TL with same parameters at all locations | –245.0 | 11.0 | 4.3×10^–4^ |
| M155 | EX at EXT/S, RL at MPA, TL at EXT/N | –245.0 | 11.0 | 4.3×10^–4^ |
| M111 | EX at EXT/N, RL at MPA, TL at EXT/S | –245.0 | 11.0 | 4.3×10^–4^ |
| M162 | RL at MPA, TL with different *λ* and same *β* and *θ* at EXT/N and EXT/S | –245.0 | 11.0 | 4.3×10^–4^ |
| M148 | RL with same *β* and different *λ* at MPA and EXT/N, TL at EXT/S | –245.0 | 11.0 | 4.2×10^–4^ |
| M211 | EX at EXT/S, TL with different *λ* and same *β* and *θ* at MPA and EXT/N | –244.9 | 11.1 | 3.9×10^–4^ |
| M233 | TL with same parameters at EXT/N and EXT/S, different at MPA | –244.9 | 11.2 | 3.9×10^–4^ |
| M173 | RL at MPA, GL with same *λ* and different *β* at EXT/N and EXT/S | –244.8 | 11.2 | 3.8×10^–4^ |
| M159 | RL with different parameters at MPA and EXT/S, TL at EXT/N | –244.8 | 11.2 | 3.8×10^–4^ |
| M154 | RW at EXT/S, RL at MPA, TL at EXT/N | –244.8 | 11.2 | 3.7×10^–4^ |
| M265 | RW at EXT/N, GL with different parameters at MPA and EXT/S | –244.7 | 11.3 | 3.7×10^–4^ |
| M207 | RW at EXT/S, TL with different *λ* and same *β* and *θ* at MPA and EXT/N | –244.7 | 11.3 | 3.6×10^–4^ |
| M147 | RL with same *λ* and different *β* at MPA and EXT/N, TL at EXT/S | –244.6 | 11.4 | 3.5×10^–4^ |
| M215 | RL at EXT/S, TL with different *λ* and same *β* and *θ* at MPA and EXT/N | –244.6 | 11.4 | 3.4×10^–4^ |
| M287 | EX at EXT/S, TL at EXT/N, GL at MPA | –244.6 | 11.4 | 3.4×10^–4^ |
| M270 | EX at EXT/N, TL at EXT/S GL at MPA | –244.6 | 11.4 | 3.4×10^–4^ |
| M291 | TL with different *λ* and same *β* and *θ* at EXT/N and EXT/S, GL at MPA | –244.6 | 11.5 | 3.4×10^–4^ |
| M304 | EX at EXT/S, GL with different parameters at MPA and EXT/N | –244.3 | 11.7 | 3.0×10^–4^ |
| M286 | RW at EXT/S, TL at EXT/N, GL at MPA | –244.3 | 11.7 | 3.0×10^–4^ |
| M288 | RL at EXT/S, TL at EXT/N, GL at MPA | –244.3 | 11.7 | 2.9×10^–4^ |
| M300 | RW at EXT/S, GL with different parameters at MPA and EXT/N | –244.2 | 11.8 | 2.8×10^–4^ |
| M130 | RL with same *λ* at MPA and EXT/N, different at EXT/S, different *β* at each location | –244.2 | 11.8 | 2.8×10^–4^ |
| M164 | RL at MPA, TL at EXT/N, GL at EXT/S | –244.2 | 11.8 | 2.8×10^–4^ |
| M244 | TL with different *λ* and same *β* and *θ* at MPA and EXT/N, GL at EXT/S | –244.0 | 12.0 | 2.6×10^–4^ |
| M308 | RL at EXT/S, GL with different parameters at MPA and EXT/N | –244.0 | 12.0 | 2.6×10^–4^ |
| M210 | EX at EXT/S, TL with same *λ* and different *β* and *θ* at MPA and EXT/N | –243.9 | 12.1 | 2.4×10^–4^ |
| M206 | RW at EXT/S, TL with same *λ* and different *β* and *θ* at MPA and EXT/N | –243.7 | 12.3 | 2.2×10^–4^ |
| M214 | RL at EXT/S, TL with same *λ* and different *β* and *θ* at MPA and EXT/N | –243.5 | 12.5 | 2.0×10^–4^ |
| M271 | EX at EXT/N, GL with same *λ* and *β* at MPA and EXT/S | –243.4 | 12.6 | 1.9×10^–4^ |
| M272 | EX at EXT/N, GL with same *λ* and different *β* at MPA and EXT/S | –243.3 | 12.7 | 1.8×10^–4^ |
| M142 | RL with same parameters at MPA and EXT/S, different at EXT/N | –243.3 | 12.7 | 1.8×10^–4^ |
| M167 | RL with same parameters at MPA and EXT/S, GL at EXT/N | –243.3 | 12.7 | 1.8×10^–4^ |
| M124 | RL with same parameters at MPA and EXT/N, different at EXT/S | –243.2 | 12.8 | 1.7×10^–4^ |
| M332 | GL with same *λ* at MPA and EXT/S, different at EXT/N, same *β* at EXT/N and EXT/S, different at MPA | –243.2 | 12.8 | 1.7×10^–4^ |
| M141 | RL with different *λ* at each location, same *β* at EXT/N and EXT/S, different at MPA | –243.0 | 13.0 | 1.5×10^–4^ |
| M243 | TL with same *λ* and different *β* and *θ* at MPA and EXT/N, GL at EXT/S | –243.0 | 13.1 | 1.5×10^–4^ |
| M137 | RL with same parameters at EXT/N and EXT/S, different at MPA | –242.8 | 13.2 | 1.4×10^–4^ |
| M146 | RL with same parameters at MPA and EXT/N, TL at EXT/S | –242.2 | 13.8 | 1.0×10^–4^ |
| M225 | TL with same *λ* at MPA and EXT/N, different at EXT/S, same *β* and *θ* at MPA and EXT/S, diff. at EXT/N | –242.0 | 14.0 | 9.5×10^–5^ |
| M274 | EX at EXT/N, GL with different parameters at MPA and EXT/S | –242.0 | 14.0 | 9.3×10^–5^ |
| M212 | EX at EXT/S, TL with different parameters at MPA and EXT/N | –241.9 | 14.1 | 9.0×10^–5^ |
| M192 | EX at EXT/N, TL with different parameters at MPA and EXT/S | –241.9 | 14.1 | 9.0×10^–5^ |
| M208 | RW at EXT/S, TL with different parameters at MPA and EXT/N | –241.9 | 14.1 | 8.9×10^–5^ |
| M161 | RL at MPA, TL with same *λ* and different *β* and *θ* at EXT/N and EXT/S | –241.8 | 14.2 | 8.4×10^–5^ |
| M171 | RL at MPA, TL at EXT/S, GL at EXT/N | –241.7 | 14.3 | 8.2×10^–5^ |
| M149 | RL with different parameters at MPA and EXT/N, TL at EXT/S | –241.7 | 14.3 | 8.2×10^–5^ |
| M216 | RL at EXT/S, TL with different parameters at MPA and EXT/N | –241.4 | 14.6 | 7.0×10^–5^ |
| M281 | RL at EXT/N, TL at EXT/S, GL at MPA | –241.3 | 14.7 | 6.7×10^–5^ |
| M290 | TL with same *λ* and different *β* and *θ* at EXT/N and EXT/S, GL at MPA | –241.3 | 14.7 | 6.7×10^–5^ |
| M310 | TL at EXT/S, GL with same *λ* and different *β* at MPA and EXT/N | –241.3 | 14.7 | 6.7×10^–5^ |
| M237 | TL with different *λ* at each location, same *β* and *θ* at EXT/N and EXT/S, different at MPA | –241.2 | 14.8 | 6.3×10^–5^ |
| M220 | TL with same parameters at MPA and EXT/N, different at EXT/S | –240.9 | 15.1 | 5.5×10^–5^ |
| M245 | TL with different parameters at MPA and EXT/N, GL at EXT/S | –240.8 | 15.2 | 5.2×10^–5^ |
| M282 | RL at EXT/N, GL with same *λ* and *β* at MPA and EXT/S | –240.8 | 15.2 | 5.1×10^–5^ |
| M229 | TL with same *λ* at MPA and EXT/S, different at EXT/N, same *β* and *θ* at all locations | –240.7 | 15.3 | 5.0×10^–5^ |
| M329 | GL with same parameters at EXT/N and EXT/S, different at MPA | –240.6 | 15.4 | 4.8×10^–5^ |
| M283 | RL at EXT/N, GL with same *λ* and different *β* at MPA and EXT/S | –240.5 | 15.5 | 4.5×10^–5^ |
| M156 | RL with same parameters at MPA and EXT/S, TL at EXT/N | –240.5 | 15.5 | 4.4×10^–5^ |
| M223 | TL with same *λ* at all locations, different *β* and *θ* at each location | –240.3 | 15.7 | 4.0×10^–5^ |
| M333 | GL with different *λ* at each location, same *β* at EXT/N and EXT/S, different at MPA | –239.9 | 16.1 | 3.2×10^–5^ |
| M322 | GL with same *λ* at MPA and EXT/N, different at EXT/S, different *β* at each location | –239.7 | 16.3 | 3.0×10^–5^ |
| M050 | RW at EXT/N, EX with same *λ* at MPA and EXT/S | –239.1 | 16.9 | 2.2×10^–5^ |
| M057 | EX with same *λ* at all locations | –239.1 | 16.9 | 2.2×10^–5^ |
| M285 | RL at EXT/N, GL with different parameters at MPA and EXT/S | –239.0 | 17.0 | 2.1×10^–5^ |
| M203 | RL at EXT/N, TL with different parameters at MPA and EXT/S | –238.4 | 17.6 | 1.6×10^–5^ |
| M252 | TL with different parameters at MPA and EXT/S, GL at EXT/N | –238.4 | 17.6 | 1.5×10^–5^ |
| M334 | GL with same parameters at MPA and EXT/S, different at EXT/N | –238.3 | 17.7 | 1.5×10^–5^ |
| M163 | RL at MPA, TL with different parameters at EXT/N and EXT/S | –238.2 | 17.8 | 1.4×10^–5^ |
| M312 | TL at EXT/S, GL with different parameters at MPA and EXT/N | –238.1 | 17.9 | 1.3×10^–5^ |
| M293 | TL at EXT/N, GL with same *λ* and *β* at MPA and EXT/S | –238.0 | 18.0 | 1.3×10^–5^ |
| M331 | GL with same *λ* at EXT/N and EXT/S, different at MPA, different *β* at each location | –237.9 | 18.1 | 1.2×10^–5^ |
| M335 | GL with same *λ* at MPA and EXT/S, different at EXT/N, different *β* at each location | –237.9 | 18.2 | 1.2×10^–5^ |
| M292 | TL with different parameters at EXT/N and EXT/S, GL at MPA | –237.8 | 18.2 | 1.1×10^–5^ |
| M238 | TL with same parameters at MPA and EXT/S, different at EXT/N | –237.6 | 18.4 | 1.0×10^–5^ |
| M218 | TL with same *λ* at all locations, same *β* and *θ* at MPA and EXT/N, different at EXT/S | –237.5 | 18.5 | 1.0×10^–5^ |
| M294 | TL at EXT/N, GL with same *λ* and different *β* at MPA and EXT/S | –237.5 | 18.5 | 9.9×10^–6^ |
| M048 | RW at EXT/N and EXT/S with same variance, EX at MPA | –237.5 | 18.5 | 9.8×10^–6^ |
| M049 | RW at EXT/N and EXT/S with different variance, EX at MPA | –237.5 | 18.5 | 9.8×10^–6^ |
| M055 | RW at EXT/S, EX with same *λ* at MPA and EXT/N | –237.5 | 18.6 | 9.7×10^–6^ |
| M337 | GL with different parameters at each location | –237.4 | 18.6 | 9.4×10^–6^ |
| M051 | RW at EXT/N, EX with different *λ* at MPA and EXT/S | –237.1 | 18.9 | 8.1×10^–6^ |
| M058 | EX with same *λ* at MPA and EXT/N, different at EXT/S | –237.1 | 18.9 | 8.0×10^–6^ |
| M060 | EX with same *λ* at EXT/N and EXT/S, different at MPA | –236.9 | 19.1 | 7.5×10^–6^ |
| M059 | EX with same *λ* at MPA and EXT/S, different at EXT/N | –236.9 | 19.1 | 7.2×10^–6^ |
| M052 | RW at EXT/N, EX at MPA, RL at EXT/S | –236.3 | 19.7 | 5.6×10^–6^ |
| M062 | EX with same *λ* at MPA and EXT/N, RL at EXT/S | –236.3 | 19.7 | 5.5×10^–6^ |
| M313 | GL with same parameters at all locations | –236.2 | 19.8 | 5.2×10^–6^ |
| M071 | EX at MPA, RL with same parameters at EXT/N and EXT/S | –236.2 | 19.8 | 5.1×10^–6^ |
| M054 | RW at EXT/N, EX at MPA, Gopertz–logistic dynamics at EXT/S | –236.0 | 20.0 | 4.7×10^–6^ |
| M066 | EX with same *λ* at MPA and EXT/N, GL at EXT/S | –236.0 | 20.0 | 4.7×10^–6^ |
| M296 | TL at EXT/N, GL with different parameters at MPA and EXT/S | –235.7 | 20.3 | 4.1×10^–6^ |
| M056 | RW at EXT/S, EX with different *λ* at MPA and EXT/N | –235.2 | 20.8 | 3.2×10^–6^ |
| M001 | RW with same variance at all locations | –235.0 | 21.0 | 2.9×10^–6^ |
| M002 | RW at all locations, with same variance at MPA and EXT/S, different at EXT/N | –235.0 | 21.0 | 2.9×10^–6^ |
| M003 | RW at all locations, with same variance at MPA and EXT/N, different at EXT/S | –235.0 | 21.0 | 2.9×10^–6^ |
| M004 | RW at all locations, with same variance at EXT/N and EXT/S, different at MPA | –235.0 | 21.0 | 2.9×10^–6^ |
| M005 | RW at all locations, with different variance at each location | –235.0 | 21.0 | 2.9×10^–6^ |
| M061 | EX at all locations, with different *λ* at each location | –234.7 | 21.3 | 2.4×10^–6^ |
| M315 | GL with same *β* at all locations, same *λ* at MPA and EXT/N, different at EXT/S | –234.6 | 21.4 | 2.4×10^–6^ |
| M006 | RW at MPA and EXT/N with same variance, EX at EXT/S | –234.5 | 21.5 | 2.2×10^–6^ |
| M007 | RW at MPA and EXT/N with different variance, EX at EXT/S | –234.5 | 21.5 | 2.2×10^–6^ |
| M091 | EX at MPA, GL with same *λ* and *β* at EXT/N and EXT/S | –234.5 | 21.5 | 2.2×10^–6^ |
| M314 | GL with same *λ* at all locations, same *β* at MPA and EXT/N, different at EXT/S | –234.5 | 21.5 | 2.2×10^–6^ |
| M069 | EX with same *λ* at MPA and EXT/S, RL at EXT/N | –234.4 | 21.6 | 2.2×10^–6^ |
| M087 | EX with same *λ* at MPA and EXT/S, GL at EXT/N | –234.4 | 21.6 | 2.1×10^–6^ |
| M180 | RW at EXT/N, TL with same parameters at MPA and EXT/S | –234.4 | 21.6 | 2.1×10^–6^ |
| M241 | TL with different parameters at each location | –234.4 | 21.6 | 2.1×10^–6^ |
| M016 | RW at MPA, EX with same *λ* at EXT/S and EXT/N | –234.4 | 21.6 | 2.1×10^–6^ |
| M063 | EX with different *λ* at MPA and EXT/N, RL at EXT/S | –233.8 | 22.2 | 1.5×10^–6^ |
| M008 | RW at MPA and EXT/N with same variance, RL at EXT/S | –233.7 | 22.3 | 1.5×10^–6^ |
| M009 | RW at MPA and EXT/N with different variance, RL at EXT/S | –233.7 | 22.3 | 1.5×10^–6^ |
| M024 | RW at MPA, RL with same parameters at EXT/N and EXT/S | –233.5 | 22.5 | 1.4×10^–6^ |
| M067 | EX with different *λ* at MPA and EXT/N, GL at EXT/S | –233.4 | 22.6 | 1.3×10^–6^ |
| M012 | RW at MPA and EXT/N with same variance, GL at EXT/S | –233.4 | 22.6 | 1.3×10^–6^ |
| M013 | RW at MPA and EXT/N with different variance, GL at EXT/S | –233.4 | 22.6 | 1.3×10^–6^ |
| M318 | GL with same *λ* at all locations, same *β* at MPA and EXT/S, different at EXT/N | –233.3 | 22.7 | 1.2×10^–6^ |
| M014 | RW at MPA and EXT/S with same variance, EX at EXT/N | –232.9 | 23.1 | 1.0×10^–6^ |
| M015 | RW at MPA and EXT/S with different variance, EX at EXT/N | –232.9 | 23.1 | 1.0×10^–6^ |
| M317 | GL with same *λ* at all locations, same *β* at EXT/N and EXT/S, different at MPA | –232.8 | 23.2 | 9.5×10^–7^ |
| M068 | RW at EXT/S, EX at MPA, RL at EXT/N | –232.8 | 23.2 | 9.5×10^–7^ |
| M086 | RW at EXT/S, EX at MPA, GL at EXT/N | –232.8 | 23.2 | 9.4×10^–7^ |
| M299 | RW at EXT/S, GL with same *β* and different *λ* at MPA and EXT/N | –232.8 | 23.2 | 9.4×10^–7^ |
| M176 | RW at EXT/N and EXT/S with same variance, TL at MPA | –232.8 | 23.2 | 9.3×10^–7^ |
| M177 | RW at EXT/N and EXT/S with different variance, TL at MPA | –232.8 | 23.2 | 9.3×10^–7^ |
| M072 | EX at MPA, RL with same *λ* and different *β* at EXT/N and EXT/S | –232.8 | 23.2 | 9.3×10^–7^ |
| M325 | GL with same *λ* at MPA and EXT/S, different at EXT/N, same *β* at all locations | –232.7 | 23.3 | 8.9×10^–7^ |
| M017 | RW at MPA, EX with different *λ* at EXT/S and EXT/N | –232.3 | 23.7 | 7.3×10^–7^ |
| M044 | RW at MPA, GL with same *λ* and *β* at EXT/N and EXT/S | –232.1 | 23.9 | 6.8×10^–7^ |
| M070 | EX with different *λ* at MPA and EXT/S, RL at EXT/N | –232.1 | 23.9 | 6.7×10^–7^ |
| M093 | EX at MPA, GL with same *β* and different *λ* at EXT/N and EXT/S | –232.1 | 23.9 | 6.6×10^–7^ |
| M088 | EX with different *λ* at MPA and EXT/S, GL at EXT/N | –232.1 | 23.9 | 6.6×10^–7^ |
| M182 | RW at EXT/N, TL with different *λ* and same *β* and *θ* at MPA and EXT/S | –232.1 | 23.9 | 6.6×10^–7^ |
| M053 | RW at EXT/N, EX at MPA, TL at EXT/S | –232.1 | 23.9 | 6.6×10^–7^ |
| M303 | EX at EXT/S, GL with same *β* and different *λ* at MPA and EXT/N | –232.1 | 23.9 | 6.6×10^–7^ |
| M178 | RW at EXT/N, EX at EXT/S, TL at MPA | –232.1 | 23.9 | 6.6×10^–7^ |
| M219 | TL with same *β* and *θ* at all locations, same *λ* at MPA and EXT/N, different at EXT/S | –232.0 | 24.0 | 6.5×10^–7^ |
| M064 | EX with same *λ* at MPA and EXT/N, TL at EXT/S | –232.0 | 24.0 | 6.5×10^–7^ |
| M227 | TL with same *λ* at EXT/N and EXT/S, different at MPA, same *β* and *θ* at all locations | –231.9 | 24.1 | 6.1×10^–7^ |
| M081 | EX at MPA, TL with same parameters at EXT/N and EXT/S | –231.9 | 24.1 | 6.1×10^–7^ |
| M186 | EX with same *λ* at EXT/N and EXT/S, TL at MPA | –231.9 | 24.1 | 6.1×10^–7^ |
| M235 | TL with same *λ* at EXT/N and EXT/S, different at MPA, different *β* and *θ* at each location | –231.9 | 24.1 | 6.0×10^–7^ |
| M078 | EX with same *λ* at MPA and EXT/S, TL at EXT/N | –231.8 | 24.2 | 5.9×10^–7^ |
| M189 | EX at EXT/N, TL with same parameters at MPA and EXT/S | –231.8 | 24.2 | 5.9×10^–7^ |
| M073 | EX at MPA, RL with same *β* and different *λ* at EXT/N and EXT/S | –231.7 | 24.3 | 5.6×10^–7^ |
| M018 | RW at MPA, EX at EXT/N, RL at EXT/S | –231.3 | 24.7 | 4.5×10^–7^ |
| M020 | RW at MPA, EX at EXT/N, GL at EXT/S | –231.0 | 25.0 | 3.9×10^–7^ |
| M089 | EX at MPA, RL at EXT/S, GL at EXT/N | –230.9 | 25.1 | 3.8×10^–7^ |
| M074 | EX at MPA, RL with different parameters at EXT/N and EXT/S | –230.9 | 25.1 | 3.8×10^–7^ |
| M307 | RL at EXT/S, GL with same *β* and different *λ* at MPA and EXT/N | –230.9 | 25.1 | 3.7×10^–7^ |
| M179 | RW at EXT/N, RL at EXT/S, TL at MPA | –230.9 | 25.1 | 3.7×10^–7^ |
| M196 | RL with same parameters at EXT/N and EXT/S, TL at MPA | –230.8 | 25.2 | 3.4×10^–7^ |
| M181 | RW at EXT/N, TL with same *λ* and different *β* and *θ* at MPA and EXT/S | –230.7 | 25.3 | 3.3×10^–7^ |
| M221 | TL with same *λ* at all locations, same *β* and *θ* at EXT/N and EXT/S, different at MPA | –230.7 | 25.3 | 3.2×10^–7^ |
| M076 | EX at MPA, RL at EXT/N, GL at EXT/S | –230.6 | 25.4 | 3.2×10^–7^ |
| M021 | RW at MPA and EXT/S with same variance, RL at EXT/N | –230.6 | 25.4 | 3.2×10^–7^ |
| M022 | RW at MPA and EXT/S with different variance, RL at EXT/N | –230.6 | 25.4 | 3.2×10^–7^ |
| M039 | RW at MPA and EXT/S with same variance, GL at EXT/N | –230.6 | 25.4 | 3.2×10^–7^ |
| M040 | RW at MPA and EXT/S with different variance, GL at EXT/N | –230.6 | 25.4 | 3.2×10^–7^ |
| M184 | RW at EXT/N, TL at MPA, GL at EXT/S | –230.6 | 25.4 | 3.2×10^–7^ |
| M094 | EX at MPA, GL with different parameters at EXT/N and EXT/S | –230.5 | 25.5 | 3.0×10^–7^ |
| M025 | RW at MPA, RL with same *λ* and different *β* at EXT/N and EXT/S | –230.4 | 25.6 | 2.9×10^–7^ |
| M092 | EX at MPA, GL with same *λ* and different *β* at EXT/N and EXT/S | –230.2 | 25.8 | 2.6×10^–7^ |
| M077 | RW at EXT/S, EX at MPA, TL at EXT/N | –230.2 | 25.8 | 2.6×10^–7^ |
| M185 | RW at EXT/S, EX at EXT/N, TL at MPA | –230.2 | 25.8 | 2.6×10^–7^ |
| M023 | RW at MPA, EX at EXT/S, RL at EXT/N | –229.9 | 26.2 | 2.2×10^–7^ |
| M046 | RW at MPA, GL with same *β* and different *λ* at EXT/N and EXT/S | –229.8 | 26.2 | 2.2×10^–7^ |
| M041 | RW at MPA, EX at EXT/N, GL at EXT/S | –229.8 | 26.2 | 2.1×10^–7^ |
| M010 | RW at MPA and EXT/N with same variance, TL at EXT/S | –229.8 | 26.2 | 2.1×10^–7^ |
| M011 | RW at MPA and EXT/N with different variance, TL at EXT/S | –229.8 | 26.2 | 2.1×10^–7^ |
| M034 | RW at MPA, TL with same parameters at EXT/N and EXT/S | –229.7 | 26.3 | 2.0×10^–7^ |
| M026 | RW at MPA, RL with same *β* and different *λ* at EXT/N and EXT/S | –229.6 | 26.5 | 1.9×10^–7^ |
| M079 | EX with different *λ* at MPA and EXT/S, TL at EXT/N | –229.3 | 26.7 | 1.6×10^–7^ |
| M191 | EX at EXT/N, TL with different *λ* and same *β* and *θ* at MPA and EXT/S | –229.3 | 26.7 | 1.6×10^–7^ |
| M230 | TL with different *λ* at each location, same *β* and *θ* at all locations | –229.3 | 26.7 | 1.6×10^–7^ |
| M065 | EX with different *λ* at MPA and EXT/N, TL at EXT/S | –229.3 | 26.7 | 1.6×10^–7^ |
| M187 | EX with different *λ* at EXT/N and EXT/S, TL at MPA | –229.3 | 26.7 | 1.6×10^–7^ |
| M083 | EX at MPA, TL with different *λ* and same *β* and *θ* at EXT/N and EXT/S | –229.3 | 26.7 | 1.6×10^–7^ |
| M253 | TL at MPA, GL with same *λ* and *β* at EXT/N and EXT/S | –229.1 | 26.9 | 1.5×10^–7^ |
| M222 | TL with same *λ* at all locations, same *β* and *θ* at MPA and EXT/S, different at EXT/N | –229.0 | 27.0 | 1.5×10^–7^ |
| M200 | RL at EXT/N, TL with same parameters at MPA and EXT/S | –229.0 | 27.0 | 1.5×10^–7^ |
| M249 | TL with same parameters at MPA and EXT/S, GL at EXT/N | –229.0 | 27.0 | 1.4×10^–7^ |
| M042 | RW at MPA, RL at EXT/N, GL at EXT/S | –228.7 | 27.3 | 1.2×10^–7^ |
| M027 | RW at MPA, RL with different parameters at EXT/N and EXT/S | –228.7 | 27.3 | 1.2×10^–7^ |
| M029 | RW at MPA, RL at EXT/N, GL at EXT/S | –228.4 | 27.6 | 1.1×10^–7^ |
| M047 | RW at MPA, GL with different parameters at EXT/N and EXT/S | –228.3 | 27.7 | 1.0×10^–7^ |
| M045 | RW at MPA, GL with same *λ* and different *β* at EXT/N and EXT/S | –228.2 | 27.8 | 9.6×10^–8^ |
| M030 | RW at MPA and EXT/S with same variance, TL at EXT/N | –228.2 | 27.8 | 9.5×10^–8^ |
| M031 | RW at MPA and EXT/S with different variance, TL at EXT/N | –228.2 | 27.8 | 9.5×10^–8^ |
| M224 | TL with same *λ* at MPA and EXT/N, different at EXT/S, same *β* and *θ* at EXT/N and EXT/S, diff. at MPA | –228.1 | 27.9 | 8.8×10^–8^ |
| M228 | TL with same *λ* at EXT/N and EXT/S, different at MPA, same *β* and *θ* at MPA and EXT/N, diff. at EXT/S | –228.0 | 28.0 | 8.8×10^–8^ |
| M080 | EX at MPA, RL at EXT/S, TL at EXT/N | –228.0 | 28.0 | 8.4×10^–8^ |
| M188 | EX at EXT/N, RL at EXT/S, TL at MPA | –228.0 | 28.0 | 8.4×10^–8^ |
| M327 | GL with same *λ* at MPA and EXT/S, different at EXT/N, same *β* at MPA and EXT/N, different at EXT/S | –227.9 | 28.2 | 8.0×10^–8^ |
| M326 | GL with different *λ* at each location, same *β* at all locations | –227.8 | 28.2 | 7.9×10^–8^ |
| M316 | GL with same parameters at MPA and EXT/N, different at EXT/S | –227.8 | 28.2 | 7.8×10^–8^ |
| M231 | TL with same *λ* at MPA and EXT/S, different at EXT/N, same *β* and *θ* at MPA and EXT/N, diff. at EXT/S | –227.7 | 28.3 | 7.4×10^–8^ |
| M190 | EX at EXT/N, TL with same *λ* and different *β* and *θ* at MPA and EXT/S | –227.7 | 28.3 | 7.4×10^–8^ |
| M236 | TL with same *λ* at MPA and EXT/S, different at EXT/N, same *β* and *θ* at EXT/N and EXT/S, diff. at MPA | –227.7 | 28.3 | 7.4×10^–8^ |
| M085 | EX at MPA, TL at EXT/N, GL at EXT/S | –227.6 | 28.4 | 7.2×10^–8^ |
| M193 | EX at EXT/N, TL at MPA, GL at EXT/S | –227.6 | 28.4 | 7.1×10^–8^ |
| M194 | RW at EXT/S, RL at EXT/N, TL at MPA | –227.4 | 28.6 | 6.4×10^–8^ |
| M246 | RW at EXT/S, TL at MPA, GL at EXT/N | –227.4 | 28.6 | 6.3×10^–8^ |
| M032 | RW at MPA, EX at EXT/S, TL at EXT/S | –227.3 | 28.8 | 5.9×10^–8^ |
| M019 | RW at MPA, EX at EXT/N, TL at EXT/S | –227.3 | 28.8 | 5.9×10^–8^ |
| M036 | RW at MPA, TL with different *λ* and same *β* and *θ* at EXT/N and EXT/S | –227.2 | 28.8 | 5.9×10^–8^ |
| M197 | RL with same *λ* and different *β* at EXT/N and EXT/S, TL at MPA | –227.0 | 29.0 | 5.1×10^–8^ |
| M328 | GL with different *λ* at each location, same *β* at MPA and EXT/N, different at EXT/S | –226.6 | 29.4 | 4.2×10^–8^ |
| M202 | RL at EXT/N, TL with different *λ* and same *β* and *θ* at MPA and EXT/S | –226.3 | 29.7 | 3.7×10^–8^ |
| M075 | EX at MPA, RL at EXT/N, TL at EXT/S | –226.3 | 29.7 | 3.7×10^–8^ |
| M082 | EX at MPA, TL with same *λ* and different *β* and *θ* at EXT/N and EXT/S | –226.3 | 29.7 | 3.7×10^–8^ |
| M195 | EX at EXT/S, RL at EXT/N, TL at MPA | –226.3 | 29.7 | 3.7×10^–8^ |
| M255 | TL at MPA, GL with same *β* and different *λ* at EXT/N and EXT/S | –226.3 | 29.7 | 3.6×10^–8^ |
| M251 | TL with different *λ* and same *β* and *θ* at MPA and EXT/S, GL at EXT/N | –226.3 | 29.7 | 3.6×10^–8^ |
| M090 | EX at MPA, TL at EXT/S, GL at EXT/N | –226.3 | 29.7 | 3.6×10^–8^ |
| M247 | EX at EXT/S, TL at MPA, GL at EXT/N | –226.3 | 29.7 | 3.6×10^–8^ |
| M311 | TL at EXT/S, GL with same *β* and different *λ* at MPA and EXT/N | –226.3 | 29.7 | 3.6×10^–8^ |
| M234 | TL with same *λ* at EXT/N and EXT/S, different at MPA, same *β* and *θ* at MPA and EXT/S, diff. at EXT/N | –226.1 | 29.9 | 3.3×10^–8^ |
| M198 | RL with same *β* and different *λ* at EXT/N and EXT/S, TL at MPA | –225.9 | 30.1 | 3.1×10^–8^ |
| M033 | RW at MPA, RL at EXT/S, TL at EXT/S | –225.9 | 30.1 | 3.0×10^–8^ |
| M038 | RW at MPA, TL at EXT/N, GL at EXT/S | –225.6 | 30.4 | 2.6×10^–8^ |
| M232 | TL with different *λ* at each location, same *β* and *θ* at MPA and EXT/N, different at EXT/S | –224.7 | 31.3 | 1.7×10^–8^ |
| M248 | RL at EXT/S, TL at MPA, GL at EXT/N | –224.7 | 31.3 | 1.6×10^–8^ |
| M199 | RL with different parameters at EXT/N and EXT/S, TL at MPA | –224.7 | 31.3 | 1.6×10^–8^ |
| M028 | RW at MPA, RL at EXT/N, TL at EXT/S | –224.5 | 31.5 | 1.5×10^–8^ |
| M035 | RW at MPA, TL with same *λ* and different *β* and *θ* at EXT/N and EXT/S | –224.5 | 31.5 | 1.5×10^–8^ |
| M201 | RL at EXT/N, TL with same *λ* and different *β* and *θ* at MPA and EXT/S | –224.4 | 31.6 | 1.5×10^–8^ |
| M043 | RW at MPA, TL at EXT/N, GL at EXT/S | –224.4 | 31.6 | 1.4×10^–8^ |
| M250 | TL with same *λ* and different *β* and *θ* at MPA and EXT/S, GL at EXT/N | –224.4 | 31.6 | 1.4×10^–8^ |
| M254 | TL at MPA, GL with same *λ* and different *β* at EXT/N and EXT/S | –224.4 | 31.6 | 1.4×10^–8^ |
| M204 | RL at EXT/N, TL at MPA, GL at EXT/S | –224.4 | 31.6 | 1.4×10^–8^ |
| M256 | TL at MPA, GL with different parameters at EXT/N and EXT/S | –224.3 | 31.7 | 1.3×10^–8^ |
| M240 | TL with different *λ* at each location, same *β* and *θ* at MPA and EXT/S, different at EXT/N | –223.0 | 33.0 | 7.2×10^–9^ |
| M084 | EX at MPA, TL with different parameters at EXT/N and EXT/S | –223.0 | 33.0 | 7.2×10^–9^ |
| M297 | RW at EXT/S, GL with same *λ* and *β* at MPA and EXT/N | –222.3 | 33.7 | 5.0×10^–9^ |
| M324 | GL with same *λ* at EXT/N and EXT/S, different at MPA, same *β* at MPA and EXT/N, different at EXT/S | –222.0 | 34.0 | 4.4×10^–9^ |
| M323 | GL with same *λ* at EXT/N and EXT/S, different at MPA, same *β* at all locations | –221.8 | 34.2 | 3.9×10^–9^ |
| M037 | RW at MPA, TL with different parameters at EXT/N and EXT/S | –221.4 | 34.6 | 3.2×10^–9^ |
| M301 | EX at EXT/S, GL with same *λ* and *β* at MPA and EXT/N | –221.2 | 34.8 | 2.8×10^–9^ |
| M305 | RL at EXT/S, GL with same *λ* and *β* at MPA and EXT/N | –219.7 | 36.3 | 1.3×10^–9^ |
| M239 | TL with same *λ* at MPA and EXT/S, different at EXT/N, different *β* and *θ* at each location | –219.3 | 36.7 | 1.1×10^–9^ |
| M226 | TL with same *λ* at MPA and EXT/N, different at EXT/S, different *β* and *θ* at each location | –217.6 | 38.4 | 0.5×10^–9^ |
| M320 | GL with same *λ* at MPA and EXT/N, different at EXT/S, same *β* at EXT/N and EXT/S, different at MPA | –217.5 | 38.5 | 0.5×10^–9^ |
| M309 | TL at EXT/S, GL with same *λ* and *β* at MPA and EXT/N | –215.8 | 40.2 | 0.2×10^–9^ |
